# Clinical features and surgical outcomes of fibrolamellar hepatocellular carcinoma: retrospective analysis of a single-center experience

**DOI:** 10.1186/s12957-020-01855-2

**Published:** 2020-05-12

**Authors:** Anastasia Lemekhova, Daniel Hornuss, Georgios Polychronidis, Philipp Mayer, Christian Rupp, Thomas Longerich, Karl-Heinz Weiss, Markus Büchler, Arianeb Mehrabi, Katrin Hoffmann

**Affiliations:** 1 Department of General, Visceral, and Transplantation Surgery, Ruprecht Karls University Hospital, Im Neuenheimer Feld 110, 69120 Heidelberg, Germany; 2 Liver Cancer Centre Heidelberg (LCCH), Heidelberg, Germany; 3 Department of Gastroenterology and Hepatology, Ruprecht Karls University Hospital, Heidelberg, Germany; 4 Department of Diagnostic and Interventional Radiology, Ruprecht Karls University Hospital, Heidelberg, Germany; 5 Institute of Pathology, University Hospital Heidelberg, Heidelberg, Germany

**Keywords:** Fibrolamellar hepatocellular carcinoma, FL-HCC, Paraneoplastic, Thromboembolism, Surgical outcome, Human, Thrombocytopenia, Hepatocellular carcinoma

## Abstract

**Background:**

Clinicopathological features and surgical outcomes of patients with fibrolamellar hepatocellular carcinoma (FL-HCC) are underreported. The aim of this study is to describe clinical characteristics and surgical outcomes for patients with this rare tumor to raise awareness among clinicians and surgeons.

**Methods:**

Retrospective review of records of a tertiary referral center and specialized liver unit was performed. Out of 3623 patients who underwent liver resection, 366 patients received surgical treatment for HCC; of them, eight (2.2%) had FL-HCC and were resected between October 2001 and December 2018.

**Results:**

Eight patients (3 males and 5 females) with FL-HCC (median age 26 years) underwent primary surgical treatment. All patients presented with unspecific symptoms or were diagnosed as incidental finding. No patient had cirrhosis or other underlying liver diseases. Coincidentally, three patients (37.5%) had a thromboembolic event prior to admission. The majority of patients had BCLC stage C and UICC stage IIIB/IVA; four patients (50%) presented with lymph node metastases. The median follow-up period was 33.5 months. The 1-year survival was 71.4%, and 3-year survival was 57.1%. Median survival was at 36.4 months. Five patients (62.5%) developed recurrent disease after a median disease-free survival of 9 months. Two patients (25.0%) received re-resection.

**Conclusion:**

FL-HCC is a rare differential diagnosis of liver masses in young patients. Since the prognosis is limited, patients with incidental liver tumors or lesions with suspicious features in an otherwise healthy liver should be presented at a specialized hepatobiliary unit. Thromboembolism might be an early paraneoplastic symptom and needs to be elucidated further in the context of FL-HCC.

## Background

Malignant primary liver tumors are rare in young adults. However, fibrolamellar hepatocellular carcinoma (FL-HCC) is an underestimated differential diagnosis of liver masses in young patients that can often be misinterpreted as benign lesions on radiological imaging and requires histopathological confirmation. FL-HCC was first described in 1956 [1] as a subtype of HCC. It has a low incidence (0.02 per 100,000) [2] and comprises between 1 and 9% of all HCC diagnoses [2–4]. The available data shows a minor male predominance (male to female case ratio 1.7:1) [2].

FL-HCC does not have specific symptoms and often presents as an incidental finding [5, 6]. In contrast to conventional HCC, it is not associated with cirrhosis, hepatitis, or other liver diseases [7, 8]. Unlike hepatic adenoma, FL-HCC has not been linked to estrogen or other hormones. Due to some radiomorphological similarities with focal nodular hyperplasia (FNH) (both may present with a stellate central scar), FL-HCC can be misinterpreted as FNH [9]. A non-negligible portion of patients presents with advanced disease of FL-HCC, but their medical history reports show prior surveillance for FNH. Typically, they are referred to tertiary centers after the growth behavior of the lesion changed or intra-hepatic metastases have occurred. FNH has been reported as a synchronous or metachronous lesion in patients with FL-HCC [10–14]. It was initially suspected as a potential precursor lesion [9, 14, 15], but causality could not be proven [16, 17]. Until proven to be a benign lesion by biopsy, every FNH with uncommon radiological characteristics such as calcifications surrounded by hypervascular features [18, 19] should be considered a potential FL-HCC. On histological evaluation, vascular invasion is often present and up to 40% of patients have already developed regional lymph node metastasis [20, 21]. Most patients are in an advanced TNM stage at the time of diagnosis [20]. Genetically, FL-HCC is defined by a focal deletion leading to DNAJB1-PRKACA gene, which can be reliably detected in formalin-fixed, paraffin-embedded tissue and is pathognomonic for FL-HCC [17, 22–24]. Immunostaining typically reveals coexpression of CD68 (KP-1 clone) and CK7 [17].

A more favorable prognosis for FL-HCC compared to classic HCC was discussed after partial hepatectomy. The 5-year survival rates in surgical reports range from 70 to 76% [4, 25] with a median overall survival between 84 and 112 months [25, 26]. In contrast, non-resectable FL-HCC showed a dismal prognosis with 5-year survival of 0% and overall median survival of 12 months [26, 27]. However, even after resection, aggressive behavior with early relapse (median time to recurrence 10–33 months [27]) and recurrence rates between 33 and 100% have been reported [4]. This underlines the absolute necessity for early detection to allow potentially curative treatment in specialized hepatobiliary units. The aim of this study was to describe clinical characteristics and surgical outcomes for patients with this rare tumor to raise awareness among clinicians and surgeons.

## Methods

This study was reviewed and approved by the ethics committee of the Medical Faculty Heidelberg at Ruprecht Karls University in Heidelberg and conducted in accordance with the Declaration of Helsinki and its subsequent amendments [28]. A retrospective analysis of patients referred to the Department of General, Visceral and Transplantation Surgery of Ruprecht Karls University for liver surgery between October 2001 and December 2018 was performed. A total of 3623 patients underwent liver resection for various conditions during this period. A total of 366 patients received a liver resection due to HCC, and of these, eight patients underwent an exploration due to FL-HCC, seven received a liver resection with primary curative intention, and one patient had an extensively metastasized intraoperative finding, rendering curative resection unattainable.

Prior to operation, each patient received a standard clinical work-up, including thorax and abdominal imaging (contrast-enhanced CT and/or MRI with liver-specific contrast agent), laboratory work-up, and clinical assessment. Clinicopathological features are shown in Table 1.

**Table 1:** Population demographics

Population demographics	
Age: median [years] (range [years])	27 (19–36)
Male/female [*n* (%)]	4/3 (57.1%/42.9%)
H/o liver disease [*n*]	–
H/o thromboembolic event [*n* (%)]	3 (42.9%)
Diabetes [*n*]	–
Alcohol abuse [*n*]	–
Smoker [*n* (%)]	2 (28.6%)
BMI median [kg/m^2^] (range [kg/m^2^])	21.90 (18.90–29.37)
Thrombocytes median [/nl] (range [/nl])	415 (255–574)
GOT/GPT: median [U/l] (range [U/l])	51/59 (20–90/20–108)
gGT/AP: median [U/l] (range [U/l])	35/102 (23–75/80–172)
INR: median [%] (range)	1.02 (0.93–1.5)
AFP > 8 IU/ml [*n* (%)]	3 (42.9%)
CEA > 2.5 μg/l [*n*]	0
CA19-9 > 37 U/ml [*n*]	0

Morbidity and mortality of the surgical procedure as well as recurrence rate and survival were analyzed as outcome parameters.

None of the patients presented with synchronous malignancies. All patients received primarily surgical treatment, and no preoperative radiological intervention (preoperative portal or hepatic vein embolization or neoadjuvant treatment) was administered. All patients received a postoperative consultation by an oncologist with recommendation in accordance with the actual recommendation.

Anatomic disease extent was described using the pTNM classification developed by Union for International Cancer Control (UICC) and the American Joint Committee on Cancer (AJCC), and clinical stage was described according to the Barcelona Clinic Liver Cancer (BCLC) classification. Liver resections are defined according to the Brisbane 2000 Terminology of liver anatomy and resections.

### Statistical analysis

Descriptive statistics were used for continuous variables; medians and range are reported. Frequency distribution described categorical variables. Mortality is defined as death occurring in the hospital or within 30 days after surgery. Due to the small sample size, analysis is limited to descriptive statistics.

## Results

Retrospective analysis identified eight patients with FL-HCC, who underwent exploration with curative intent within a 17-year period (October 2001to December 2018). Five female and three male patients with a median age of 26 years (range 18–36 years) received surgery.

Three patients had a liver mass as incidental finding on imaging prompted by unrelated conditions: one patient received abdominal MRI after an X-ray performed for shoulder pain complaint showed elevated diaphragm on the right side, one patient received abdominal ultrasound after presenting at the hospital with DVT, and one patient received a CT scan of the thorax due to pneumonia, which revealed a mass in the observable section of the liver. Four patients had vague abdominal discomfort that led to imaging and diagnosis.

All patients presented with the absence of liver disease, and no cirrhosis was found on pathology examinations of the non-tumor tissue.

AFP was slightly elevated (> 8 IU/ml) in two patients (25.0%); however, no patient had an elevation above 15 IU/ml. Preoperative laboratory findings were unremarkable in all patients and showed no liver dysfunction. Pathological findings are presented in Table 2. 62.5% presented with BCLC stage C, and the rest (37.5%) had stage A.

**Table 2:** Tumor characteristics

Tumor histopathologic features	
Number of lesions	
Single [*n* (%)]	6 (85.7%)
Multiple [*n* (%)]	1 (14.3%)
Median size [cm (range)]	13 (3.5–24)
Nodal metastasis [*n* (%)]	3 (42.9%)
Metastasis [*n* (%)]	1 (14.3%)
Vascular invasion [*n* (%)]	5 (71.4%)
Microvascular invasion [*n* (%)]	3 (42.9%)
Macrovascular invasion [*n* (%)]	2 (28.6%)
UICC stage	
I [*n*]	–
II [*n*]	–
III A [*n*]	–
III B [*n* (%)]	3 (37.5%)
III C [*n* (%)]	–
IV A [*n* (%)]	3 (37.5%)
IV B [*n* (%)]	2 (25.0%)

Median time between diagnosis through biopsy or imaging and surgery was 21 days with a range of 7 to 240 days.

Incidentally, three patients (37.5%) had a thromboembolic event prior to admission. Otherwise, no patient presented with major preoperative morbidity, such as cardiovascular, pulmonary, or metabolic diseases.

**Fig. 1:**
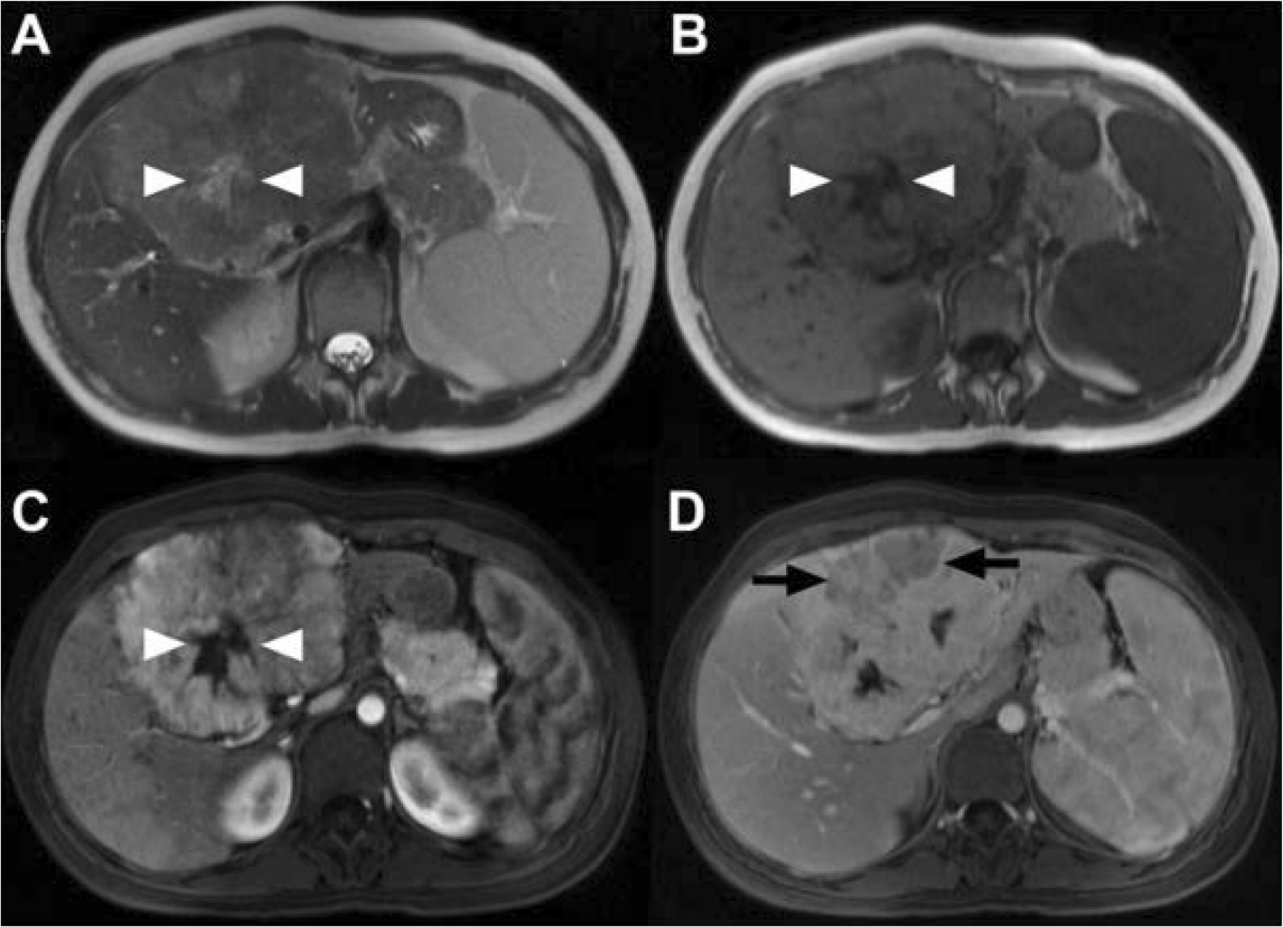
MRI scan of a 17-year-old female patient. **a**) Axial T2 weighted Half-Fourier Acquisition Single-shot Turbo spin Echo (HASTE) sequence shows a large inhomogenous hepatic lesion with a central moderately T2-hyperintense scar (white arrowheads) in the left lateral and medial as well as right anterior sectors. **b**) The central scar (white arrowheads) is more prominent in the axial native T1 Fast Low-Angle Shot (Flash) 2D sequence. **c**) The lesion shows markedly inhomogenous hyperenhancement in the arterial phase (axial T1 FLASH 3D). The central scar (white arrowheads) does not enhance. **d**) A ventral part of the lesion shows washout appearance (black arrows) in the portal venous phase (axial T1 FLASH 3D).

Imaging showed well-circumscribed lesions with a central scar, and most showed an arterial hyperenhancement. Figure 1 exemplifies findings in the current group.

Seven patients (85.7%) who underwent curative treatment presented with a single lesion, and one patient had multiple (12.5%).

Three patients (37.5%) received major resections, and four (50.0%) were treated with minor resection (typical or atypical resection of three segments or less). One patient scheduled for curative surgery showed prior undiagnosed extensive peritoneal metastasis at exploration and received an open biopsy, which confirmed the diagnosis.

There was no in-house or 90-day mortality among this group of patients. Postoperative morbidity rate was 25% for major complications (≥ Clavien-Dindo grade 3) and involved two patients. One patient developed multiple complications: a bile leak (grade C), early postoperative portal vein thrombosis, hematothorax, and ARDS; one patient had a wound dehiscence requiring repeated surgery.

One patient underwent resection at our center due to recurrence after being surgically treated with a right hemihepatectomy 2 years prior at another hospital, after which the patient was monitored for recurrence.

Median follow-up was 47 months (range 1 to 60 months). The recurrence rate after hepatectomy was 71.4 % (intrahepatic: *n* = 3, diffuse: *n* = 2). Five patients (62.5%) received treatment for progressive disease: either as chemotherapy alone or in combination with radiation or local radiological therapies as an individual approach. Three patients have died during follow-up. The 1-year survival was 71.4%, and 3-year survival was 57.1%, with a median survival time of 36.5 months after resection. Follow-up is summarized in Table 3.

**Table 3:** Follow-up summary

Patient	Extent of surgery	Recurrence	Localization of recurrence	Time to recurrence	Treatment for recurrence/progressive disease	Current status	Follow-up period
1	Biopsy	No (initial peritoneal metastasis)			Sorafenib	DOD	6 months
2	Atypical resection segments 2/3 and 4b	No				NED	57 months
3	Anatomical resection segments 2/3	Yes	Multiple intrahepatic	9 months	Resection, cisplatin/gemcitabine, sorafenib, study regimen (oral FGF401 vs. oral FGF401 with PDR001)	AWD	47 months
4	Meso-hepatectomy 4a and 4b plus segment 1 resection	Yes	Intrahepatic	30 months	Re-resection	AWD	60 months
5	Anatomical resection segments 2/3	Yes	Intrahepatic	14 months	Radiotherapy, TACE, SIRT	AWD	53 months
6	Left trisectionectomy with 4/5 en-block gastrectomy	No				NED	1 month
7	Right trisectionetomy	Yes	Intrahepatic, lymph node, pulmonary, peritoneal	3 months	Study regimen (lenvatinib vs. sorafenib)	DCU	20 months
8	Left hemihepatectomy	Yes	Peritoneal	4 months	Pembrolizumab	DOD	7 months

*NED* no evidence of disease, *AWD* alive with disease, *DOD* dead of disease, *DCU* dead, cause unknown

## Discussion

After FL-HCC was first described by Edmondson in 1956, little progress has been made in the diagnosis and treatment of this entity. Especially in the European region, there are only limited reports available, thus making even an estimation of incidence for the region difficult. Most reports are limited to case descriptions. In the USA, FL-HCC estimates less than 1% of all primary liver tumors according to the SEER database [3]; in contrast, Mexico reported an incidence rate of 5.8% [29]. Table 4 provides an overview of the reports on clinicopathological features and treatments of FL-HCC from the European region published in the last 10 years.

**Table 4:** Recent reports on fibrolamellar hepatocellular carcinoma from the European region (2009–2019)

Report	Number of patients	Age (y)	Male/female	Initial clinical features	Stage	Number of lesions	Vascular invasion	Positive lymph nodes	Resection margin	Chemotherapy	Resection	Recurrence after surgery	OS
Ince et al. [46]	1	19	0/1	Abdominal pain	NR	Mult	Microvasc	Yes	–	No	Unresectable	–	26 m
Bill et al. [47]	1	28	1/0	Abdominal pain	NR	Mult	NR	NR	–	Palliative: sorafenib, doxorubicin, everolimus	Unresectable	–	23 m
Mafeld et al. [44]	1	52	0/1	Abdominal discomfort	NR	1	Macrovasc	NR	R0	Neoadjuvant: TACE, SIRT (Y-90)	Resected	–	NR
Ciurea et al. [48]	1	23	0/1	Abdominal pain, distended lower abdomen	NR	Mult	NR	NR	R0	Adjuvant: cisplatin, 5-FU; sorafenib	Resected	Yes, at 26 m	61 m
Estrella Diez et al. [49]	1	16	1/0	Weight loss, jaundice, abdominal pain	NR	1	NR	NR	–	Oxaliplatin, folinate calcium, 5-FU	Unresectable	–	NR
Bauer et al. [50]	1	29	1/0	Incidental finding	NR	1	NR	NR	NR	Adjuvant: sorafenib	Resected	Yes, within 24 m	NR
Bender et al. [51]	1	19	0/1	Elevated liver enzymes	NR	NR	NR	NR	NR	Sorafenib, bevacizumab, erlotinib, platinum, doxorubicin, gemcitabine	Resected	Yes, NR	NR
Vandewynckel et al. [52]	1	26	1/0	NR	NR	NR	NR	NR	–	Palliative: cisplatin, doxorubicin, sorafenib	Unresectable	–	NR
Sulaiman and Geberhiwot [53]	1	14	0/1	NR	NR	NR	NR	NR	NR	Adjuvant, sorafenib	Resected	Yes, within 3 y	6 y
Chiarelli et al. [54]	1	62	1/0	NR	NR	1	NR	NR	NR	No	Resected	No, FU 36 m	NR
Okur et al. [55]	1	12	0/1	Weight loss, constipation, fatigue	NR	3	NR	Yes	R0	Neoadjuvant: cisplatin, doxorubicin Adjuvant: cyclophosphamide, thalidomide; 5-FU, IFN-α	Resected	Yes, at 21 m	NR
Zen et al. [56]	14	Median 19 (range 11–38)	6/8	NR	NR	NR	NR	NR	NR	No	Resected	NR	NR
Minutolo et al. [57]	1	29	1/0	RUQ pain, nausea, vomiting	NR	1	NR	NR	Rx (ruptured)	Adjuvant: NR	Resected	Yes, at 6 m	26 m
De Gaetano et al. [58]	1	25	1/0	Abdominal pain, obstructive jaundice	NR	1	NR	No	R0	No	Resected	No, FU 36 m	NR
Berger et al. [59]	1	22	0/1	Weight loss, constipation, vomiting	NR	Mult	NR	Yes	–	Yes: bleomycin, etoposide, cisplatin	Unresectable	–	< 1 m
Wojcicki et al. [60]	1	28	0/1	NR	NR	1	NR	Yes	NR	No	Resected	Yes, at 23 m	114 m
Gras et al. [61]	1	25	0/1	Mass of the right hypochondrium	NR	1	NR	NR	NR	Adjuvant: gemcitabine, oxaliplatin	Resected	Yes, at 6 m	39 m
Malouf et al. [30]	40	Median 22 (range 9–65)	9 (22%)/31 (78%)	Abdominal pain (55%), weight loss (25%), hepatomegaly	AJCC: I (67%) II (0%) III (23%) IV (10%)	1 (90%), mult (10%)	Microvasc (52%)	Yes (27%)	NR	Adjuvant (48%)	Resected	23 pts within 7.8 y	18 pts in 7.8 y
Benito et al. [45]	1	26	0/1	NR	NR	1	NR	NR	NR	Adjuvant: sunitinib	Resected	No, at 12 m	12 m
Koudah et al. [62]	1	24	1/0	RUQ pain, weight loss	NR	1	NR	NR	NR	NR	Resected	NR	NR
Brunel et al. [63]	1	22	0/1	Abdominal pain, fever, palpable mass	NR	1	NR	No	NR	NR	Resected	No, at 25 m	25 m
Mroz et al. [64]	1	28	1/0	Dyspnea, cough, hemoptysis, chest pain, fever, general weakness, left leg pain	NR	Mult.	NR	NR	NR	5-FU, cisplatin, doxorubicin	Unresectable	–	NR
Terzis et al. [65]	1	23	1/0	Abdominal discomfort	NR	1	Macrovasc	NR	No	No	Unresectable	NR	NR

*5-FU* 5-fluorouracil, *NR* not reported, *m* months, *y* years, *pts* patients, *FU* follow-up, *mult* multiple, *RUQ* right upper quadrant

FL-HCC predominantly affects younger patients, ages 10–30 years old, although a second incidence peak has been described at ages 60–69 [2]. The current series consisted of patients all aged below 36 years all presented with pure FL-HCC. It is conceivable and should be evaluated further, if patients falling into the second incidence peak have a fibrolamellar-like, conventional HCC. A systematic testing for a DNAJB1-PRKACA fusion gene is needed to assess this.

The slight male predominance reported in the analysis of the SEER database [2] is consistent with the current dataset. Most patients either present with incidental findings or undergo work-up for abdominal pain and weight loss [30], and the current dataset reports consistent findings.

Three patients had a history of deep venous thrombosis, with one showing acute signs, due to extensive phlebothrombosis, spreading to the inferior vena cava. Although venous thromboembolism is associated with a number of cancers [31], it is not commonly described for HCC, especially rare HCC subtypes. There is an established association between cirrhosis and venous thromboembolism [32, 33], but cirrhosis in FL-HCC patients is uncommon. A link between thrombocytosis as paraneoplastic syndrome of HCC due to TPO-overproduction and large tumor volume, as well as high alpha-fetoprotein, has previously been described by Hwang et al. [34]. Two patients with history of deep venous thrombosis also had thrombocytosis on admission in the current cohort, and all three had large tumor volume, although alpha-fetoprotein was fairly low (< 13.5 IU/ml) in all patients. Only few reports describe an association between FL-HCC and thrombosis, such as atrial thrombus and pulmonary emboli [35] and thrombus in the main portal vein [36]. A large-scale study is needed to further investigate the association of thrombocytosis or thromboembolic events and FL-HCC.

Other paraneoplastic symptoms have previously been described in case reports for FL-HCC. Hyperammonemic encephalopathy [37–39] is the most prevalent symptom described in the literature. Several pathophysiological pathways need to be evaluated in these cases, such as hepatocellular dysfunction, portosystemic shunting, and ornithine transcarbamylase mutation. However, in most reported cases, none of these mechanisms sufficiently explains the degree of hyperammonemic encephalopathy. Table 5 provides an overview of reports describing FL-HCC presenting with potential paraneoplastic symptoms. Interestingly, the association between FL-HCC and gynecomastia has only been described in pediatric population.

**Table 5:** Paraneoplastic symptoms reported in literature

Paraneoplastic symptom	Report
Hyperammonemic encephalopathy	Chapuy et al. [66]
	Sulaiman and Geberhiwot [53]
	Sethi et al. [67]
	Bender et al. [51]
	Hashash et al. [68]
	Alsina et al. [69]
	Berger et al. [59]
	Chan et al. [70]
	Surjan et al. [38]
	Suarez et al. [71]
	Thakral and Simonetto [39]
Venous thrombosis	Hashash et al. [68]
	Bhagat et al. [36]
	Asrani and LaRusso [35]
	Khoo and Clouston [72]
	Marrannes et al. [73]
	Lamberts et al. [74]
	Saab and Yao [75]
	Mansouri et al. [76]
	Vandewynckel et al. [52]
Gynecomastia	Muramori et al. [77]
	Smith et al. [78]
	Sher et al. [79]
	Hany et al. [80]
	McCloskey et al. [81]
	Agarwal et al. [82]
	Saab and Yao [75]
Cold agglutinin disease	Al-Matham et al. [83]

Vascular invasion was present in six patients: one proved to be unresectable, while five received surgery with curative intent. Four of these patients developed a recurrence. Three patients had positive lymph nodes, two of which developed a recurrence. Vascular invasion and lymph node metastasis have been described in association with a worse outcome after surgical treatment of FL-HCC [40, 41]. The current study supports previously described association.

As with most cancers, negative resection margins are associated with a better outcome [41]. In the current dataset, five of seven successfully resected patients had negative resection margins, while two remaining had microscopically positive resection margins. Four R0 resected patients showed a recurrence, and one patient with R1 situation had a recurrence within 3 months. Despite R0 resection margins, some series report a high recurrence rate of up to 71% for FL-HCC [42]. Interestingly, four out of five R0 resected patients showed longer survival (median survival 53 months, range 7–60 months) compared to R1 resected patients, despite most presenting with a recurrence after surgery.

Female gender has been previously described as a variable associated with a better overall survival [42]; however, other reports contradict this finding [5]. Interestingly, both patients in the current dataset who died during follow-up were female.

Surgery remains the mainstream treatment for FL-HCC; however, chemotherapy does not offer potential benefit in unresectable patients. Most patients receive sorafenib in unresectable cases with unsatisfactory results. Some publications report a stable disease under this regimen [42], while others reported progression [43]. Few case reports explore non-standard treatment options with varying reports. Mafeld et al. reported a case of FL-HCC successfully treated with TACE and subsequent SIRT using Yttrium-90 leading to tumor downsize to a resectable size [44]. Benito et al. reported usage of adjuvant sunitinib after the resection of FL-HCC with metastasis to the ovary with recurrence-free patient at 12 months [45]. Doubtless, studies of chemotherapies in unresectable patients are necessary in the future.

## Conclusion

The clinicopathological features and outcomes described in this report are consistent with those published in the literature. Further reports from the European region are necessary to evaluate FL-HCC further in this part of the world. Since deep venous thrombosis is not usually present in young, otherwise healthy individuals, with no liver cirrhosis, findings in this report are thought-provoking and the association between FL-HCC and vascular thromboembolism should be studied on a larger scale. Further, more reports on adjuvant or palliative treatment of FL-HCC may shed light on chemotherapy regimens with most beneficial clinical outcomes.

Due to limited data on FL-HCC, low incidence affecting predominantly young patients without comorbidities and oftentimes vascular invasion at the time of diagnosis, clinicians should be vigilant. It is imperative to promptly refer patients with incidental liver masses to a hospital with a specialized hepatobiliary surgery unit for evaluation and surgical treatment. Due to a similar radiological presentation of FL-HCC and FNH, all patients presenting with atypical FNH on imaging should be evaluated at a tertiary referral center to avoid fatal outcome due to misdiagnosis.

## Data Availability

All data generated or analyzed during this study are included in this published article and its supplementary information files.

## Abbreviations

AJCC: American Joint Committee on Cancer
ARDS: Acute respiratory distress syndrome
BCLC: Barcelona Clinic Liver Cancer
DVT: Deep venous thrombosis
FL-HCC: Fibrolamellar hepatocellular carcinoma
FNH: Focal nodular hyperplasia
HCC: Hepatocellular carcinoma
SIRT: Selective internal radiation therapy
TACE: Transarterial chemoembolization
TAE: Transarterial embolization
UICC: Union for International Cancer Control
